# Isolation and molecular characteristics of D2 genotype of Aichivirus D in dairy cattle in China

**DOI:** 10.3389/fvets.2025.1551420

**Published:** 2025-02-19

**Authors:** Nan Yan, Dongping Xu, Hua Yue, Cheng Tang

**Affiliations:** ^1^College of Veterinary Medicine, Southwest University, Chongqing, China; ^2^College of Animal Husbandry and Veterinary Medicine, Southwest Minzu University, Chengdu, China

**Keywords:** Aichivirus D, dairy cattle, diarrhea, isolation, genome

## Abstract

Aichivirus D (AiV-D), a newly emerging member of the *Kobuvirus* genus, is associated with diarrhea in cattle. This study aimed to investigate the prevalence and molecular characteristics of AiV-D among dairy cattle in China. From October 2021 to August 2022, 279 fecal samples were collected from diarrheal dairy cattle across seven provinces in China. Among these, 37 samples (13.2%) tested positive for AiV-D by RT-PCR, indicating a wide geographical distribution of AiV-D in Chinese dairy cattle. Phylogenetic analysis based on the complete VP1 gene revealed that Chinese dairy cattle AiV-D strains belong to the AiV-D2 genotype, with unique amino acid changes in VP0, VP3, and VP1 that distinguish them from known AiV-D strains. Additionally, an AiV-D strain was successfully isolated, and its complete genome was sequenced. Phylogenetic analysis of the complete genome and individual genes confirmed the strain’s classification within the AiV-D2 genotype. This study reports the first detection of the AiV-D2 genotype outside Japan, highlighting the need for future surveillance to better understand the epidemiology and diversity of AiV-D in China.

## 1 Introduction

*Kobuviruses*, members of the *Picornaviridae* family, are associated with diarrhea in humans and animals globally (1). The *Kobuvirus* genome comprises a 5′-UTR, a large open reading frame (ORF), and a 3′-UTR. The ORF encodes structural proteins P1 (VP0, VP3, and VP1) and nonstructural proteins P2 (2A-2C) and P3 (3A-3D) (2).

A diverse range of *Kobuviruses* has been identified across various animal species, encompassing ungulates, carnivores, rodents, birds, rabbits, and bats (3–5). The International Committee on Taxonomy of Viruses (ICTV) categorizes the genus *Kobuvirus* into six distinct species, labeled Aichivirus A-F. Aichivirus A is known to infect humans, dogs, cats, and birds (6–9); Aichivirus B has been detected in cattle, sheep, and ferrets (10–12); Aichivirus C is comprised of porcine and caprine kobuviruses (13, 14); Aichivirus E and F are represented by rabbit and bat kobuviruses, respectively (15, 16); Additionally, there are three unclassified kobuviruses, including the grey squirrel kobuvirus, Norway rat kobuvirus, and bat kobuvirus.

Aichivirus D (AiV-D), first identified in 2015 from bovine diarrhea fecal samples in Japan through metagenomics (3), has since been classified into two genotypes by the ICTV: AiV-D1 and AiV-D2. These genotypes were detected only in Kagoshima Prefecture, Japan, with detection rates of 10.4 and 16.9%, respectively (3). In 2021, AiV-D was identified in sheep on the Qinghai-Tibet Plateau in China, potentially representing a novel genotype, AiV-D3, with a detection rate of 9.2% in diarrhea fecal samples from four farms across three counties (4). Building on these findings, our previous study identified a new genotype of AiV-D (AiV-D4) in yaks (17). Our study identified that the detection rate of AiV-D4 in three provinces of the Qinghai-Tibet Plateau in China was 24.8%. Based on the successful isolation of the AiV-D4 and animal infection experiment, we confirmed its role as a diarrhea-causing pathogen with a broad geographical distribution across the Qinghai-Tibet Plateau. Most recently, a recombinant AiV-D strain was reported in cattle in Italy in 2024 (18). The present study aims to investigate the prevalence and molecular characteristics of AiV-D among dairy cattle in China, contributing to the global understanding of this emerging pathogen.

## 2 Materials and methods

### 2.1 Specimen collection

A total of 279 fecal samples were collected from dairy cattle at the time of presentation with diarrhea from October 2021 to August 2022. The sample distribution was as follows: 93 from Sichuan, 49 from Nei Mongol, 35 from Ningxia, 31 from Heibei, 26 from Shanxi, 25 from Jiangsu, and 20 from Anhui. All samples were shipped on ice and stored at −80°C in our laboratory.

### 2.2 RNA extraction and cDNA synthesis

To extract viral RNA, a 20% fecal suspension was prepared in phosphate-buffered saline (PBS) and clarified by centrifugation at 12,000 × g for 15 min. RNAiso Plus^®^ (TaKaRa Bio Inc., Shiga, Japan) was employed to extract viral RNA from the clinical samples, followed by reverse transcription using the PrimeScript^™^ RT Reagent Kit (TaKaRa) according to the manufacturer’s protocol. The synthesized cDNA was stored at −20°C for subsequent analysis.

### 2.3 Screening for AiV-D in dairy cattle

AiV-D was detected using a real-time RT-PCR assay established from our previous study (17). The PCR products were purified using the Omega Gel kit (Omega), cloned into the pMD19-T vector (TaKaRa Bio Inc., Japan), and sent for Sanger sequencing (Sangon Biotech Co., Ltd., China, Shanghai).

### 2.4 Complete VP0, VP3, and VP1 gene sequences amplification

The full-length VP0, VP3, and VP1 genes were amplified from AiV-D positive samples using specific primer pairs from our previous study (17). The PCR products were purified using the Omega Gel kit (Omega), cloned into the pMD19-T vector (TaKaRa Bio Inc., Japan), and sent for Sanger sequencing (Sangon Biotech Co., Ltd., China, Shanghai).

### 2.5 Virus isolation and identification

The supernatants from all AiV-D positive samples were cultured on Vero cells at 37°C for 2 h, after which the mixtures were discarded and replaced with 4 mL of Dulbecco’s Modified Eagle’s Medium supplemented with 100 units/mL penicillin and 100 μg/mL streptomycin. If the strains were successfully isolated, the isolated strains were further purified using the plaque technique as previously described (19), and the virus titre was determined by the Reed-Muench method. The purified strain was subjected to purity testing, followed by PCR detection of bovine diarrhea pathogens such as bovine rotavirus A (BRVA), bovine coronavirus (BCoV), bovine viral diarrhea virus (BVDV), bovine nebovirus (BNeV), bovine norovirus (BNoV), bovine torovirus (BToV), salmonella, enterotoxigenic escherichia coli (ETEC) and *cryptosporidium andersoni* (*C. andersoni*). The primer information for PCR detection is provided in Supplementary Table S1. The procedures for Indirect Immunofluorescence Assay (IF) and Transmission Electron Microscopy (TEM) were conducted as previously described (17). As a note, the anti-AiV-D-VP1 polyclonal antibody was prepared by our laboratory for IF.

### 2.6 Complete genome amplification of AiV-D strain in dairy cattle

The 12 pairs of primers in our previous study were used to amplify the genome sequence from the AiV-D isolate (17). The PCR products were cloned into the pMD19-T simple vector (TaKaRa Bio Inc.), and sent for Sanger sequencing (Sangon Biotech Co., Ltd., China, Shanghai).

### 2.7 Sequence and phylogeny analyses

For the genetic analysis of the identified AiV-D strains, we utilized the SeqMan software within the DNASTAR platform (version 7.0) to assemble the sequences, and sequences were compared with those available in GenBank using BLAST. Nucleotide and deduced amino acid sequence homologies were determined using the MegAlign program of DNASTAR platform (version 7.0). Phylogenetic trees based on nucleotide sequences were constructed using the maximum likelihood method and the Kimura two-parameter model in MEGA 11.0, with bootstrap analyses based on 1,000 replicates.

## 3 Results

### 3.1 Detection the AiV-D in dairy cattle

In our study, out of the 279 diarrhea samples collected from dairy cattle across various provinces, 37 samples (13.2%) tested positive for AiV-D by RT-PCR and confirmed by sequencing. The regional distribution of positive samples included 22.9% (8/35) from Ningxia, 13.9% (13/93) from Sichuan, 12.9% (4/31) from Hebei, 12.2% (6/49) from Nei Mongol, 12.0% (3/25) from Jiangsu, 7.7% (2/26) from Shanxi, and 5.0% (1/20) from Anhui, indicating a widespread presence of AiV-D in dairy cattle populations in these regions (Table 1).

**Table 1:** Dairy cattle fecal sample collection and AiV-D detection in China.

Province	Number of samples	Number of farms	Positive rate of AiV-D	Positive rate of farms
Sichuan	93	4	13.9%(13/93)	75.0%(3/4)
Jiangsu	25	2	12.0%(3/25)	50.0%(1/2)
Shanxi	26	3	7.7%(2/26)	33.3%(1/3)
Hebei	31	2	12.9%(4/31)	100.0%(2/2)
Ningxia	35	3	22.9%(8/35)	100.0%(3/3)
Nei Mongol	49	3	12.2%(6/49)	100.0%(3/3)
Anhui	20	2	5.0%(1/20)	50.0%(1/2)
Total	279	19	13.2%(37/279)	73.6%(14/19)

### 3.2 Amplification of the VP0, VP3, and VP1 gene sequences

**Figure 1:**
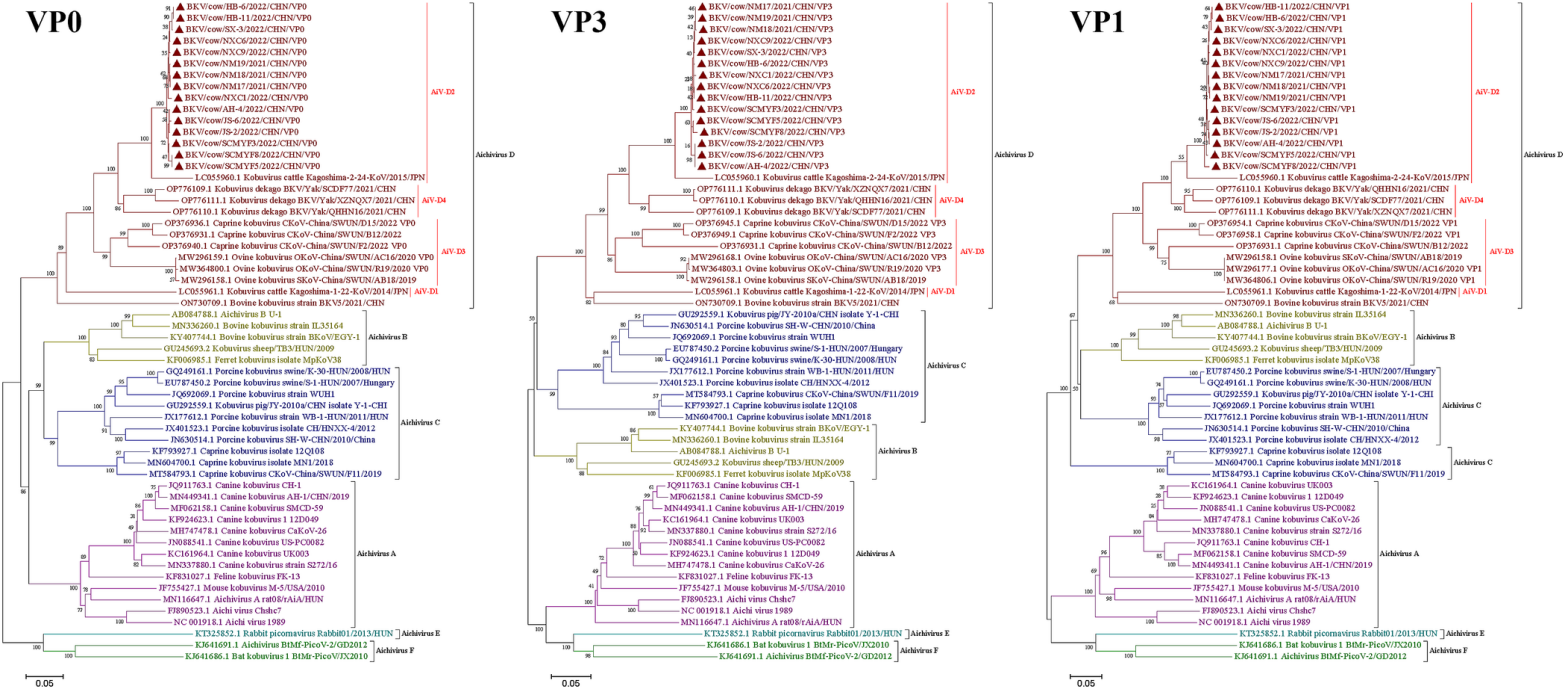
Phylogenetic trees based on complete AiV VP0, VP3, and VP1 genes. Maximum-likelihood analysis in combination with 1,000 bootstrap replicates was used to derive a phylogenetic tree based on the complete nucleotide sequences of AiV VP0, VP3, and VP1 genes. ▲represents the AiV strains from this study.

**Figure 2:**
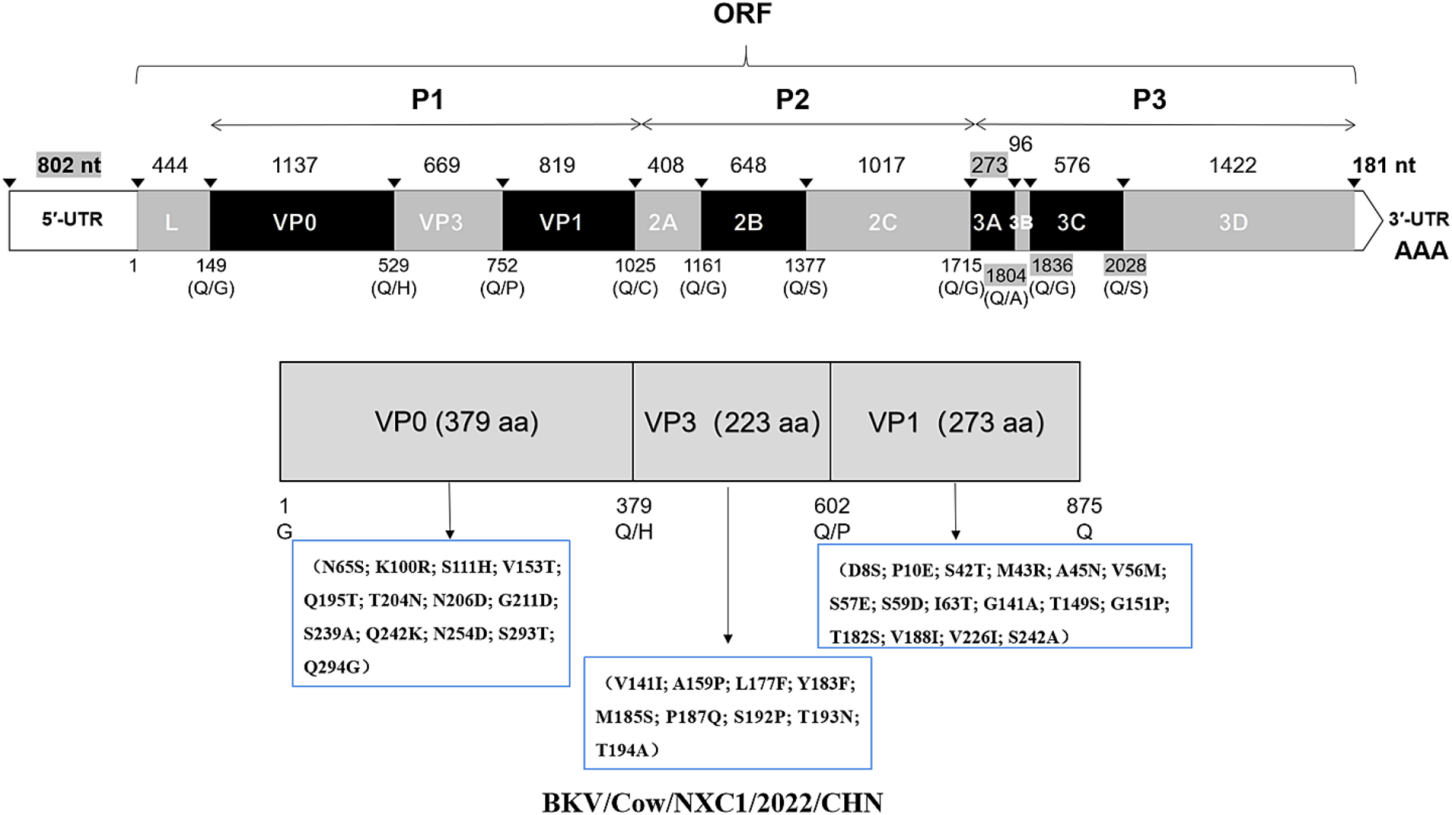
Schematic representation of the dairy cattle AiV-D genome in this study. The length of each gene is indicated by plain numbers. The differences in length between strains are indicated by gray background numbers. Black triangles show predicted cleavage sites. And a schematic diagram of unique amino acid changes in VP0, VP3, and VP1 were marked.

To further investigate the molecular characteristics of the VP0, VP3, and VP1 genes of dairy cattle AiV-D strains, all of the 37 positive samples were used for amplifying the VP0, VP3, and VP1 gene. The complete VP0, VP3, and VP1 sequences were successfully obtained from the 15 AiV-D positive samples (3 from Nei Mongol, 3 from Ningxia, 3 from Sichuan, 2 from Heibei, 2 from Jiangsu, 1 from Shanxi, and 1 from Anhui). All obtained sequences have been deposited in the GenBank (OQ267697-OQ267711). The phylogenetic tree was constructed based on the individual gene of VP0, VP3, and VP1, and all revealed that strains from this study cluster together with strain Kagoshima-2-24-KoV/2015/JPN (GenBank number LC055960) of the AiV-D2 genotype but are located on an independent branch (Figure 1). The 15 complete VP0 sequences shared 98.8–100.0% nt identity and 99.2–100.0% aa identity with each other, and shared 91.4–91.7% nt identity and 95.5–95.8% aa identity with strain Kagoshima-2-24-KoV/2015/JPN; The 15 complete VP3 sequences shared 98.4–100% nt identity and 99.6–100.0% aa identity with each other, and shared 92.1–92.8% nt identity and 95.5–96.0% aa identity with strain Kagoshima-2-24-KoV/2015/JPN; The 15 complete VP1 sequences shared 98.4–99.9% nt identity and 98.9–100.0% aa identity with each other, and shared 89.4–90.1% nt identity and 93.0–93.8% aa identity with strain Kagoshima-2-24-KoV/2015/JPN. Additionally, an amino acid comparison with the AiV-D2 genotype strain Kagoshima-2-24-KoV revealed that there were 13 unique identical aa changes in the VP0 gene (N65S; K100R; S111H; V153T; Q195T; T204N; N206D; G211D; S239A; Q242K; N254D; S293T; Q294G), 6 out of 13 aa changes located in the RBD (200–261 aa) of VP0; In VP3 gene, 15 sequences in this study shared 9 unique identical aa changes (V141I; A159P; L177F; Y183F; M185S; P187Q; S192P; T193N; T194A); In VP1 gene, there were 16 unique identical aa changes (D8S; P10E; S42T; M43R; A45N; V56M; S57E; S59D; I63T; G141A; T149S; G151P; T182S; V188I; V226I; S242A); 2 out of 16 aa changes (S42T and M43R) in the conserved antigenic peptide (22–44 aa) of VP1 (Figure 2).

### 3.3 Virus isolation and identification

**Figure 3:**
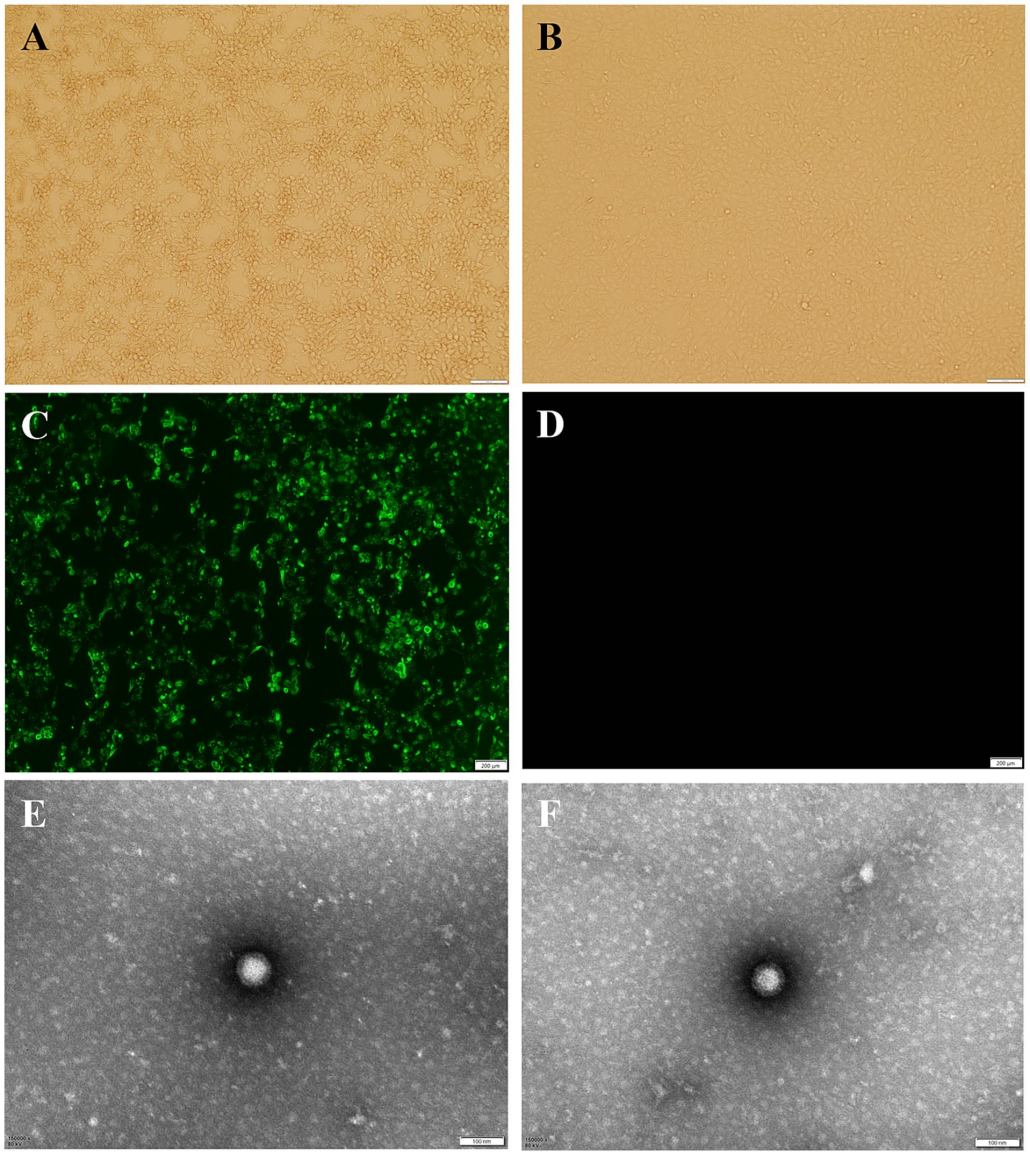
Isolation and identification of dairy cattle AiV-D2. **(A)** Vero cell infected with AiV-D2 strain showing CPE at 72 h; **(B)** Mock infection negative control Vero cell; **(C)** IF of AiV-D2 strain; **(D)** IF negative control (mock infected cells); **(E)** and **(F)** TEM image of AiV-D2 strain.

AiV-D positive samples were inoculated into Vero cell cultures and one sample out of 37 samples displayed CPE, characterized by round shrinkage, shedding and fusion at 48–72 h in passage one, with stable CPE observed about 72 h after passage 6 (Figure 3A). This isolate was plaque-purified three times at passage 6 and CPE characteristics found to be the same as before. The bovine kobuvirus isolate was designated as BKV/Cow/NXC1/2022/CHN (Cow/NXC1), and its titer was determined to be 10^–6.9^ TCID_50_/0.1 mL. The results of the purity testing on the isolated strain Cow/NXC1 revealed that all tests for bovine diarrhea pathogens were negative. The isolate was identified as AiV-D by immunofluorescence (Figure 3C); Non-enveloped, spherical virus particles (approximately 50 nm in diameter) was visualized on TEM (Figure 3E).

### 3.4 Genomic characterization of cow/NXC1

**Figure 4:**
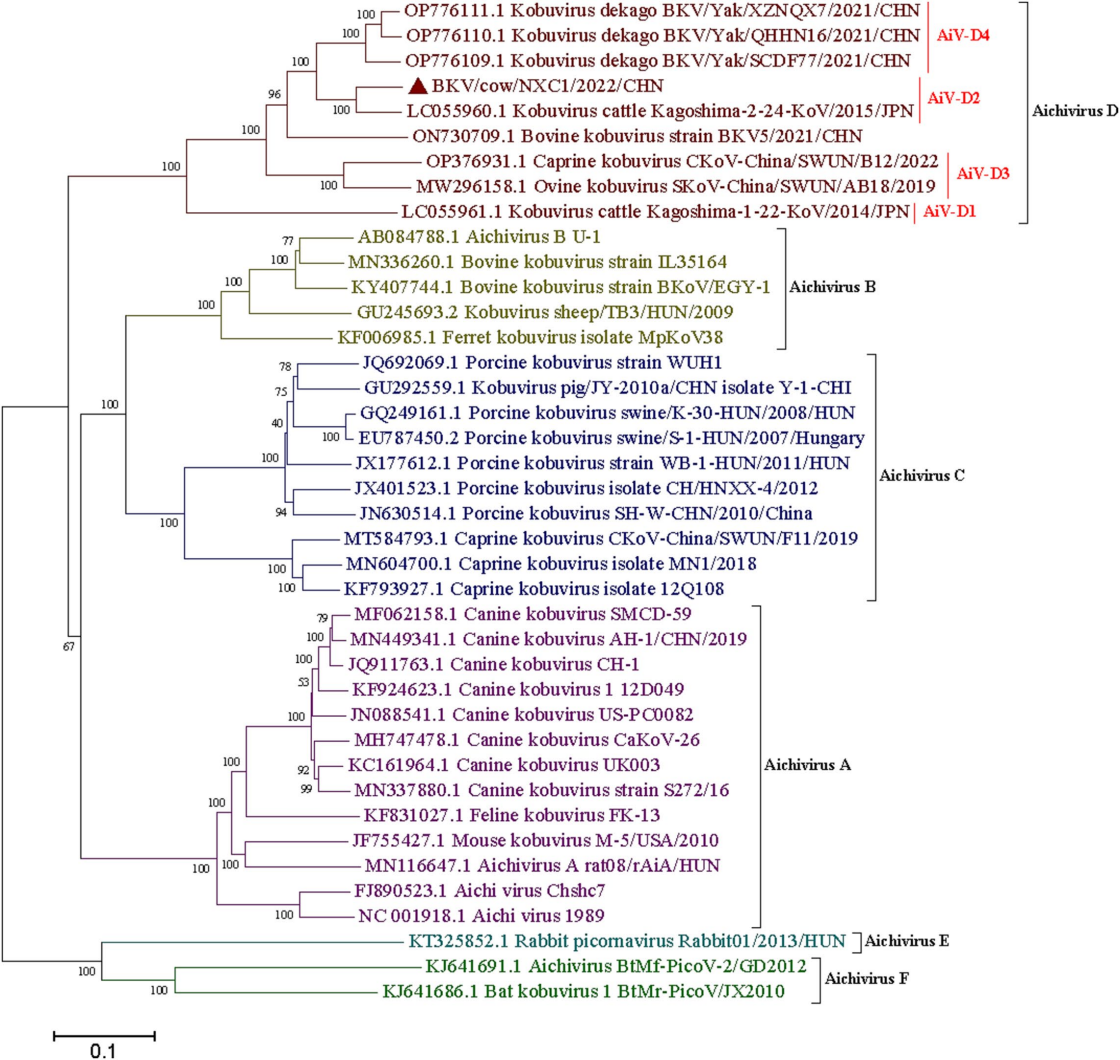
Phylogenetic tree based on the complete genomes of all AiV species **(A-F)**. Maximum-likelihood analysis in combination with 1,000 bootstrap replicates was used to derive a phylogenetic tree based on the complete genome sequences of six species AiV strains. ▲represents the AiV-D2 strain from this study.

**Figure 5:**
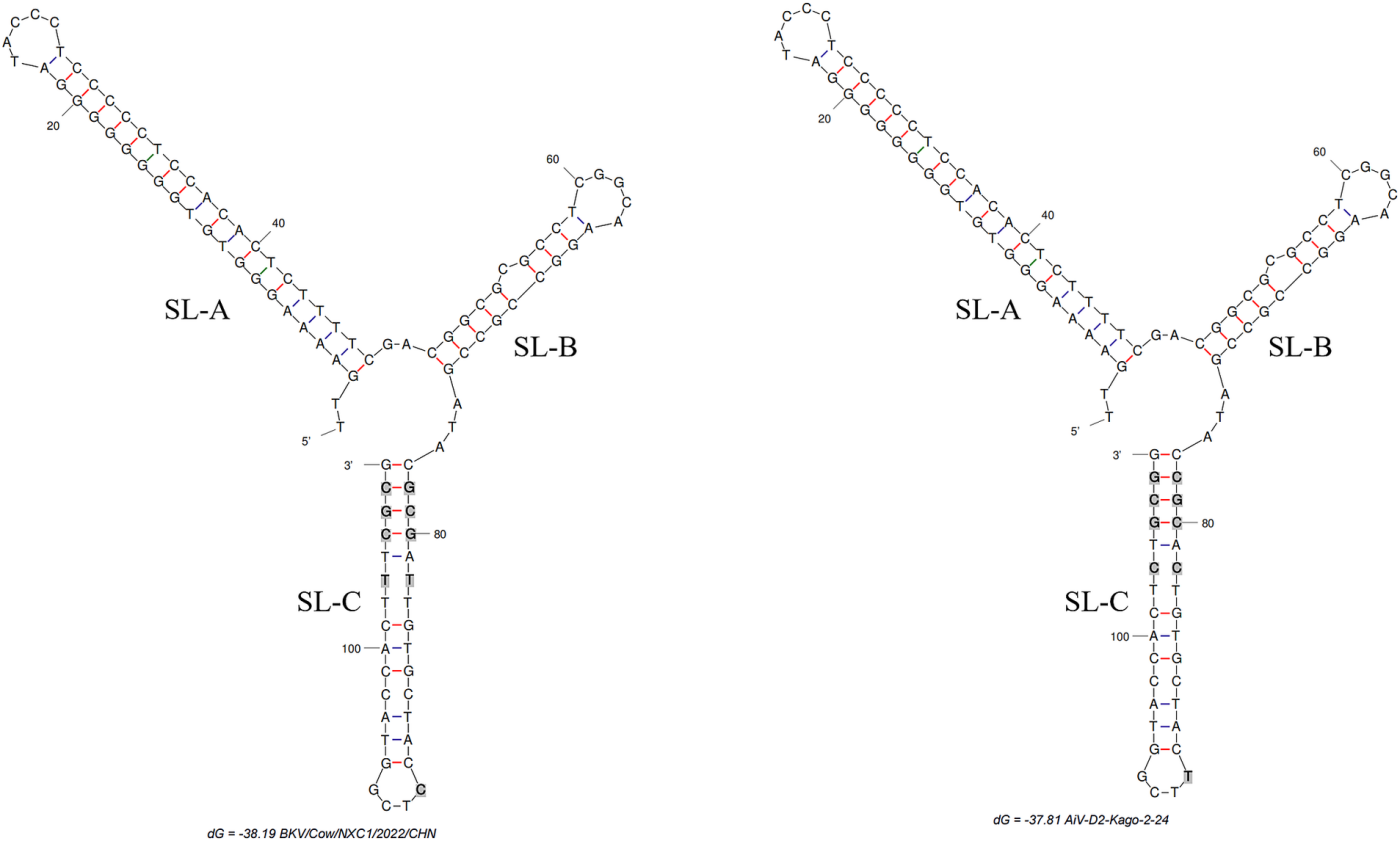
The predicted secondary structures of the 5′-UTR of the genome of AiV-D2. Bold and gray indicate differences between two strains. The positions of the 3 stem-loops are shown. Schematic of secondary structures was predicted using Mfold software (http://www.unafold.org/mfold/applications/dna-folding-form.php) and modified using RNAviz (http://rnaviz.sourceforge.Net/).

The complete genome of strain Cow/NXC1 (GenBank No.OQ267712) was obtained from the isolate. The genome was 8,492 nt in length with 55.99% GC content. This sequence had a standard *Kobuvirus* genome organization, which have a contained 7,509 bp complete ORF, which encodes a polyprotein comprising structural proteins, P1 (VP0, VP3, and VP1), and nonstructural proteins P2 (2A-2C) and P3 (3A-3D), the genomes structure map shown in Figure 2. The genome sequence of Cow/NXC1 had the highest nt and aa identities with AiV-D2 genotype strain Kagoshima-2-24-KoV/2015/JPN (GenBank No.LC055960). Phylogenetic tree based on the complete genome showed that Cow/NXC1 was grouped into AiV-D2 genotype (Figure 4). Moreover, the potential secondary structure of the 5′-UTR of the genome consists of three stem-loop domains (SL-A, SL-B, SL-C) (Figure 5). Compared with the AiV-D2 genotype strain Kago-2-24, in SL-A and SL-B, Cow/NXC1 was identical to strain Kago-2-24; in SL-C, Cow/NXC1 differed from Kago-2-24 at 9 nt positions.

## 4 Discussion

Being a newly emerging virus, knowledge of the epidemiology and molecular characteristics of AiV D remains limited. In this study, 13.2% (37 out of 279) of diarrhea fecal samples from dairy cattle tested positive for AiV-D using RT-PCR, indicating a broad geographical distribution of AiV-D in China’s dairy cattle population. Previously, AiV-D in cattle had been detected in Japan, with detection rates of 10.4 and 16.9% for AiV-D1 and AiV-D2, respectively, in 77 diarrhea fecal samples from black cattle in Kagoshima Prefecture (3). Our findings report the first detection of the AiV-D2 genotype outside Japan, highlighting its prevalence in China’s dairy cattle. These results underscore the significance for diagnosing and controlling diarrhea in dairy cattle and emphasize the need for future surveillance to enhance our understanding of the epidemiology and diversity of AiV-D in China.

The VP0 protein of Aichivirus A is potentially associated with viral pathogenesis (20). The core domain, located at amino acids 195–253 in VP0, is likely active in receptor binding (20). These motifs are conserved across the same Aichivirus species, and the VP0 gene is present in all representative Aichivirus species (4, 21). Fifteen VP0 gene sequences from dairy cattle exhibited six unique amino acid changes in the receptor-binding domain (RBD) compared to the AiV-D2 genotype strain kago-2-24. The VP3 protein of Aichivirus C (Porcine kobuvirus) inhibits the IFN-*β*-triggered signaling pathway, leading to immune evasion via the IFN signaling pathway (22). Compared to the AiV-D2 genotype strain kago-2-24, fifteen VP3 gene sequences displayed nine unique amino acid changes in the VP3 protein. Further research is warranted to explore the biological significance of these unique amino acid changes in VP0 and VP3 of AiV-D in dairy cattle.

The VP1 gene of Aichivirus A has been implicated in viral pathogenesis (23), with residues 228–240 aa (PRPPPPLPPLPTP) in VP1 of Aichivirus A recognized as a motif for binding to the enteric receptor (20). Additionally, an antigenic epitope survey revealed a highly antigenic VP1 epitope 21–43 aa (DNSPQPRTTFDYTDNPLPPDTKL), conserved among various Aichivirus strains from humans, canines, porcines, cattle, and sheep (24). The VP1 receptor-recognition motif sequences of AiV-D in dairy cattle (PRAPPTTASAPST) identified in this study were located at 227–239 aa of VP1, identical to the AiV-D2 genotype strain kago-2-24. Interestingly, the antigenic epitope motif of AiV-D in dairy cattle exhibited two combining forms identified in this study within the 22–44 aa of VP1 (VSAPETRTTFEYKDAPRPPDTRL), differing from the AiV-D2 genotype strain kago-2-24 (VSAPETRTTFEYKDAPRPPDSML). The VP1 protein is involved in neutralizing antibodies (24), and amino acid changes in the antigenic peptide region among different AiV-D strains may affect cross-protection between antibodies, potentially leading to immune escape for these strains. Therefore, monitoring the variation of the AiV-D VP1 gene is crucial for providing a theoretical basis for the development of an effective vaccine.

In our preceding research, we successfully identified the AiV-D4 genotype of Aichivirus D in yaks, establishing its role as a pathogen causing diarrhea with a wide geographical spread across the Qinghai-Tibet Plateau (17). However, the pathogenicity of other AiV-D genotype strains in cattle and sheep remains to be elucidated, and the host range of this virus warrants further exploration. The current study successfully isolated the AiV-D2 genotype strain. Although only one AiV-D2 genotype strain was isolated from 37 positive samples, this is likely due to the low viral load in the clinical samples. This result provides a foundation for further research into its biological characteristics. Additionally, the genomic identification of the isolate in dairy cattle revealed that the dairy cattle AiV-D strain was grouped into the AiV-D2 genotype based on the complete genome and individual genes. Notably, the VP0, VP3, and VP1 of the isolate exhibited unique amino acid changes distinct from the known AiV-D2 genotype strain, and the conformation of the 5′-UTR of the isolate differed from that of the known AiV-D2 genotype strain. The 5′-UTR of Kobuvirus contains three stem-loop structures (SLA, SL-B, and SL-C) that play essential roles in replication, transcription, and infectivity (17). Proper folding of the three stem-loops on the positive-strand RNA is necessary for RNA replication (25). Previous studies have indicated that the loop segment of SL-C sequences is involved in viral virulence, and mutations in the upper and lower stems of SL-C can significantly decrease viable virus yields (26). In this study, compared to the AiV-D2 genotype strain Kago-2-24, Cow/NXC1 was identical to strain Kago-2-24 in SL-A and SL-B; however, in SL-C, Cow/NXC1 had unique nucleotide changes. Further investigation is needed to determine whether the unique nucleotide changes in the 5′-UTR affect the replication and transcription of different AiV-D strains and whether they influence the virulence of the virus.

## 5 Conclusion

In summary, the present study reported the first detection of the AiV-D2 genotype outside Japan, which has been prevalent in dairy cattle in China. The molecular characteristics suggest that the dairy cattle AiV-D strains differed from the known strain in the AiV-D2 genotype. These findings have significant implications for diagnosing and controlling diarrhea in dairy cattle, highlighting a need for future surveillance of AiV-D toward a better understanding of both epidemiology and diversity of AiV-D in China.

## Data Availability

The datasets presented in this study can be found in online repositories. The names of the repository/repositories and accession number(s) can be found below: https://www.ncbi.nlm.nih.gov/genbank/, OQ267697-OQ267712.
