# Supplementary material for: Isolation and molecular characteristics of D2 genotype of Aichivirus D in dairy cattle in China
Source: Front Vet Sci. 2025 Feb 19;12:1551420. doi: 10.3389/fvets.2025.1551420 (PMC11880938; doi:10.3389/fvets.2025.1551420)
Supplement: Supplementary file 1 [file Supplementary_Table_1.DOCX]

Supplementary Material

# Supplementary Table

Table S1 PCR primers of bovine diarrhea pathogens

| Pathogens | Primers (5'-3') | length |
| --- | --- | --- |
| Bovine Rotavirus A (BRVA) | F：CCACCAGGTATGAATTGGAC | 231 bp |
|  | R：GAGTAATCACTCAGATGGCG |  |
| Bovine Coronavirus (BCoV) | F：ACWCARHTVAAYYTNAARTAYGC | 251 bp |
|  | R：TCRCAYTTDGGRTARTCCCA |  |
| Bovine Viral Diarrhea Virus (BVDV) | F：GCCATGCCCTTAGTAGGACT | 230 bp |
|  | R：CACCCTATCAGGCTGTRTYC |  |
| Bovine Nebovirus (BNeV) | F：CAGCCCGTCTGGGTGAAT | 524 bp |
|  | R：CCAGCGTTAGCGTTCCAG |  |
| Bovine Norovirus (BNoV) | F：AGTTAYTTTTCCTTYTAYGGBGA | 532 bp |
|  | R：GTGTCTCTGTCAGTCATCTTCAT |  |
| Bovine torovirus (BToV) | F：GTACTAWTTTTCCAGCTYTGC | 406 bp |
|  | R：CCAACACAAATCCGCAAYGC |  |
| Salmonella | F：AGAGTTTGATCCTGGCTCAG | 1500 bp |
|  | R：TACGGCTACCTTGTTACGACTT |  |
| Enterotoxigenic Escherichia coli (ETEC) | F：TGGGACTACCAATGCTTCTG | 450 bp |
|  | R：TATCCACCATTAGACGGAGC |  |
| Cryptosporidium andersoni (C.andersoni) | F：ATTGAACCTGAACTIGCCTA | 263 bp |
|  | R：AAAAGTCGGCAAATAACAA |  |
